# Supplementary figures and images for: Conservation of Nematocida microsporidia gene expression and host response in Caenorhabditis nematodes
Source: PLoS One. 2022 Dec 19;17(12):e0279103. doi: 10.1371/journal.pone.0279103 (PMC9762603; doi:10.1371/journal.pone.0279103)

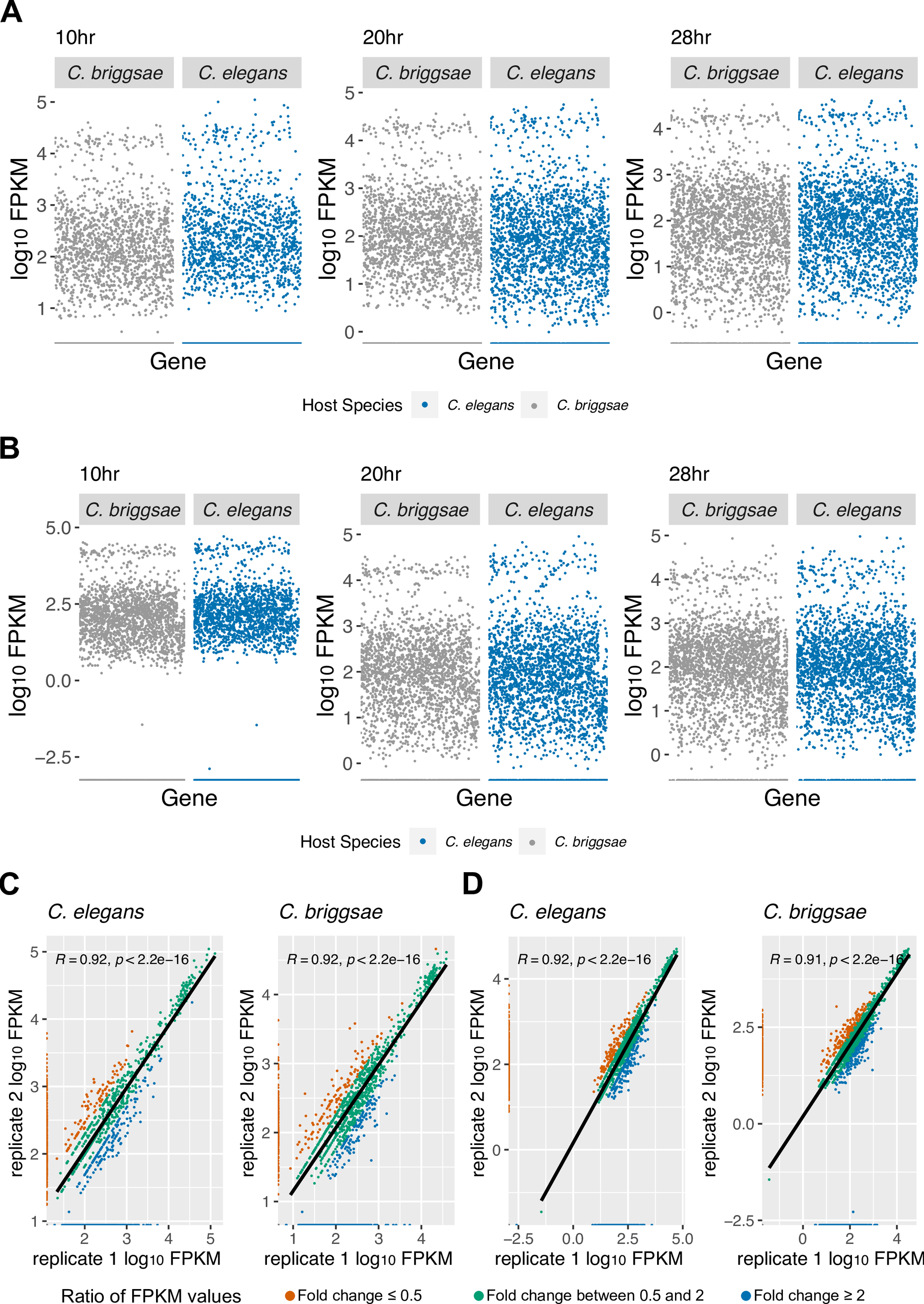

Supplement: S1 Fig — (A-B) Scatterplot of N. parisii (A) and N. ausubeli (B) log10 FPKM values between C. briggsae and C. elegans replicates at 10 hours. Pearson correlation values and p-values are indicated on the top left of each plot. The ratio of FPKM values for each gene between the replicates is demonstrated by the colour of the points. (C-D) Gene expression pattern of N. parisii (C) and N. ausubeli (D) in C. elegans and C. briggsae. (TIF) [file pone.0279103.s001.tif]

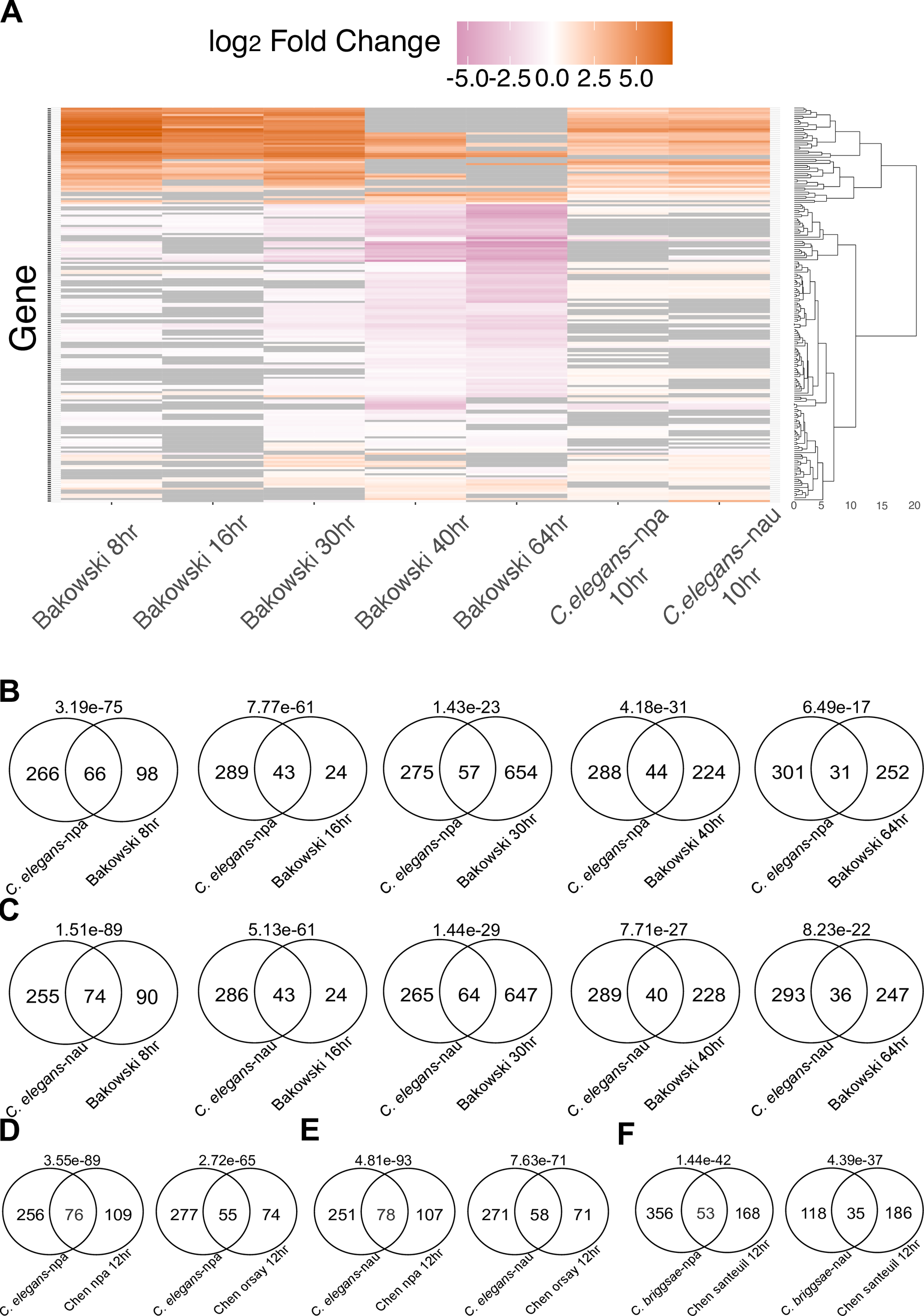

Supplement: S2 Fig — (A) Heatmap of transcriptional profiles of differentially regulated genes (FDR<0.05) across five samples from Bakowski et al. and our 10hr N. parisii and N. ausubeli infected C. elegans samples. Only genes expressed in at least four out of the eight total samples are included. (B-C) Statistical overlap of significant genes between respective N. parisii or N. ausubeli infected C. elegans samples and Bakowski et al. samples across five timepoints. (D-E) Statistical overlap of significant genes between N. parisii (D) or N. ausubeli (E) infected C. elegans samples and Chen et al. Orsay virus infected C. elegans sample at 12 hours. (F) Statistical overlap of significant genes between respective N. parisii or N. ausubeli infected C. briggsae sample and Chen et al. Santeuil virus infected C. briggsae sample at 12 hours. The p-value of each comparison is indicated on top of each Venn diagram. npa (N. parisii) and nau (N. ausubeli). (TIF) [file pone.0279103.s002.tif]

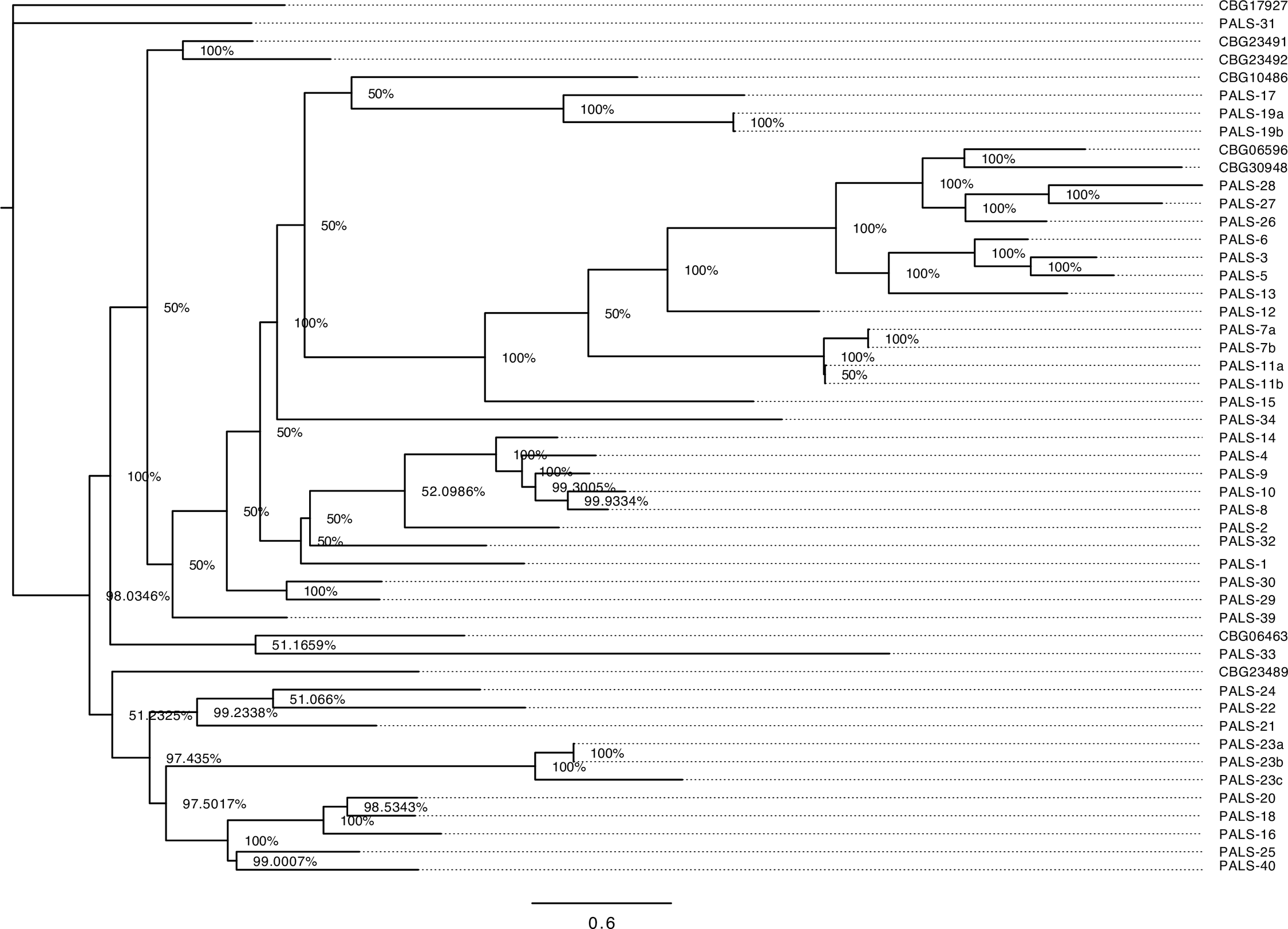

Supplement: S3 Fig — Genes with the same name that end with different letters indicate protein isoforms. Node values indicate posterior probabilities for each split as a percentage. The scale bar indicates average branch length measured in expected substitutions per site. (TIF) [file pone.0279103.s003.tif]

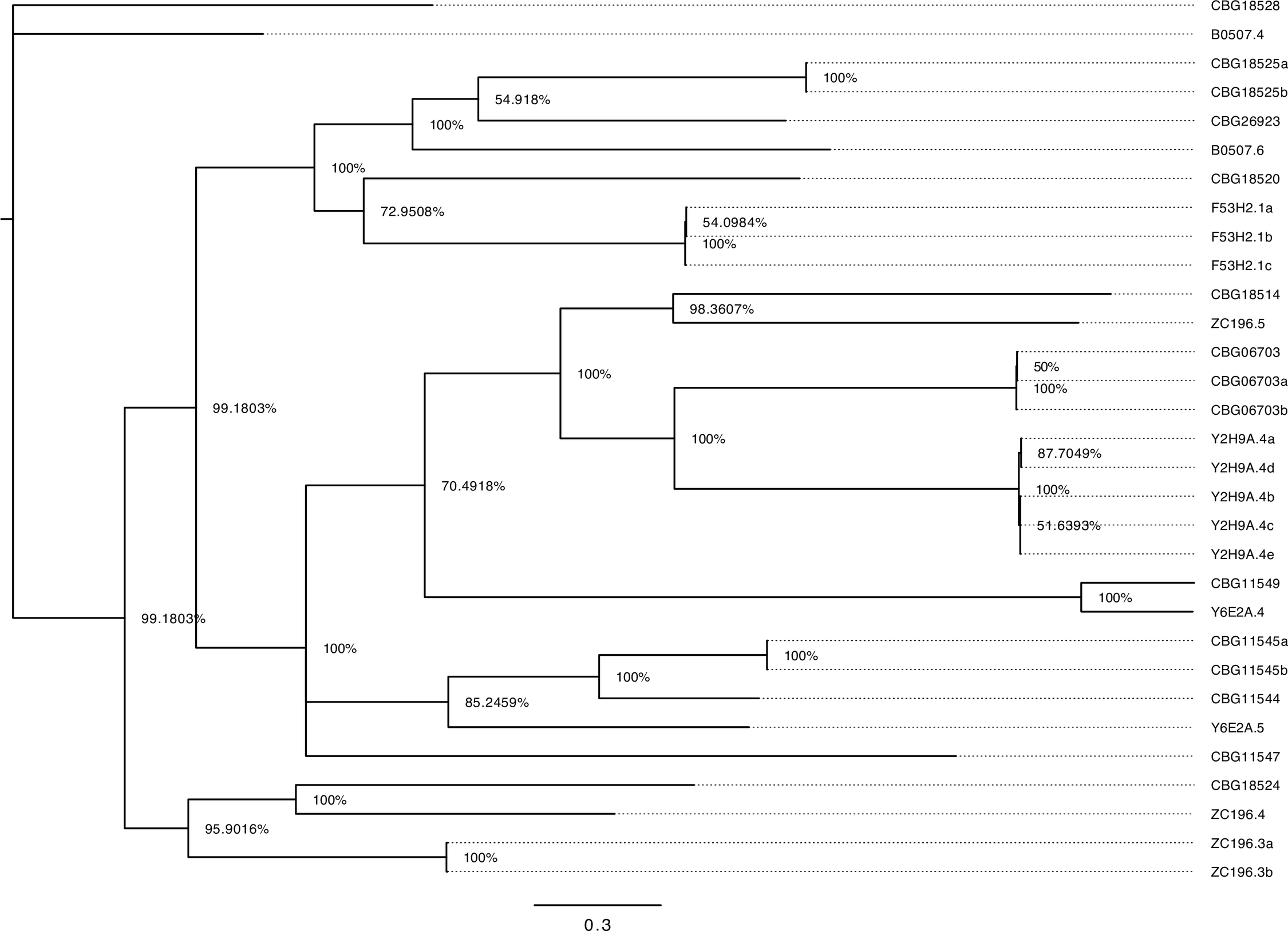

Supplement: S4 Fig — Genes with the same name that end with different letters indicate protein isoforms. Node values indicate posterior probabilities for each split as a percentage. The scale bar indicates average branch length measured in expected substitutions per site. (TIF) [file pone.0279103.s004.tif]

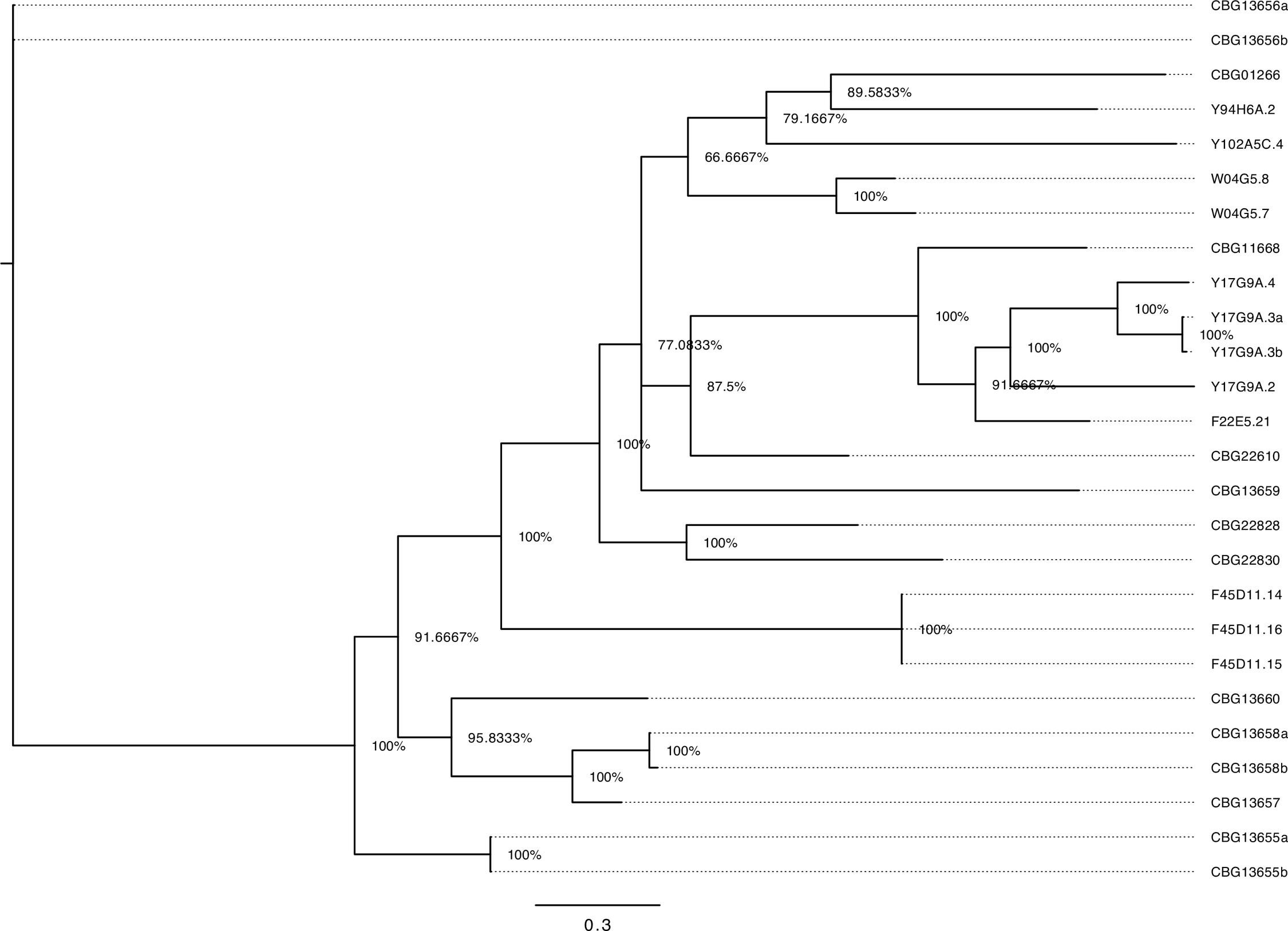

Supplement: S5 Fig — (TIF) [file pone.0279103.s005.tif]
