# Supplementary material for: Conservation of Nematocida microsporidia gene expression and host response in Caenorhabditis nematodes
Source: PLoS One. 2022 Dec 19;17(12):e0279103. doi: 10.1371/journal.pone.0279103 (PMC9762603; doi:10.1371/journal.pone.0279103)
Supplement: S6 Table — (DOCX) [file pone.0279103.s011.docx]

**S6 Table. Gene classes and domains used for enrichment analyses.**

| **Domain/ Gene Family** | **Databse** | **Wormbase gene class OR Pfam ID** |
| --- | --- | --- |
| F-box | Wormbase | fbxa, fbxb, fbxc |
| MATH (meprin or Traf homology) or BATH (BTB and MATH domain-containing) | Wormbase | math, bath |
| PALS (protein containing ALS2CR12 signature) | Wormbase | pals |
| C-type lectins | Wormbase | clec |
| DUF713 | Pfam | PF015218 |
| DUF684 | Pfam | PF05075 |
| Chil | Wormbase | chil |
| Nematode cuticle collagen N-terminal domain | Pfam | PF01484 |
| CUB and CUB-like | Pfam | PF00431, PF02408 |
| Cytochrome P450 | Wormbase | cyp |
| Glucosyltransferase family 92 | Pfam | PF00201 |
| UDP-glucuronosyltransferase | Wormbase | ugt |
| skr (Skp1-related) | Wormbase | Skr |
| Other (Any genes not in the domain or gene family above) | - | - |
